# 5-Chloro-1-[(*E*)-3-(dimethyl­amino)­acrylo­yl]-3-methyl-1*H*-benzimidazol-2(3*H*)-one–6-chloro-1-[(*E*)-3-(dimethyl­amino)­acrylo­yl]-3-methyl-1*H*-benzimid­azol-2(3*H*)-one (4/1)

**DOI:** 10.1107/S1600536811024706

**Published:** 2011-06-30

**Authors:** Rachida Dardouri, Yousef Kandri Rodi, Natalie Saffon, El Mokhtar Essassi, Seik Weng Ng

**Affiliations:** a Laboratoire de Chimie Organique Appliquée, Faculté des Sciences et Techniques, Université Sidi Mohamed Ben Abdallah, Fés, Morocco; b Service Commun Rayons-X FR2599, Université Paul Sabatier Bâtiment 2R1, 118 route de Narbonne, Toulouse, France; c Laboratoire de Chimie Organique Hétérocyclique, Pôle de Compétences Pharmacochimie, Université Mohammed V-Agdal, BP 1014 Avenue Ibn Batout, Rabat, Morocco; d Department of Chemistry, University of Malaya, 50603 Kuala Lumpur, Malaysia

## Abstract

In the reaction of 7-chloro-1,5-benzodiazepine-2,4-dione with *N*,*N*-dimethyl­formamide/dimethyl­acetal, the diazepine seven-membered ring undergoes a contraction to form the five-membered ring. The reaction yields two isomers the title compound, C_13_H_14_ClN_3_O_2_; the major component has the chlorine-atom substituent in the 5-position of the benzimidazolone ring and the minor component has the chlorine atom in the 6-position. The two isomers form a disordered co-crystal, the chloro­methyl­benzimidazolone portion of both components are disordered with respect to each other in a 4:1 ratio [the refined ratio is 0.816 (5):0.184 (5)]; the dimethyl­amino­cryloyl substitutent is ordered. The double bond of the dimethyl­amino­acryloyl substituent has an *E* configuration.

## Related literature

For the structure of the 7-chloro-1,5-benzodiazepine-2,4-dione reactant, see: Mondieig *et al.* (2007 ▶). 
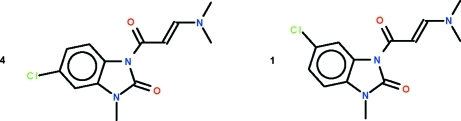


## Experimental

### 

#### Crystal data

C_13_H_14_ClN_3_O_2_
*M* *_r_* = 279.72
Orthorhombic, 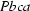

*a* = 7.3145 (2) Å
*b* = 14.2903 (3) Å
*c* = 25.1512 (6) Å
*V* = 2628.96 (11) Å^3^
*Z* = 8
Mo *K*α radiation
μ = 0.29 mm^−1^
*T* = 293 K
0.30 × 0.25 × 0.05 mm

#### Data collection

Bruker APEXII diffractometer
Absorption correction: multi-scan (*SADABS*; Sheldrick, 1996 ▶) *T*_min_ = 0.918, *T*_max_ = 0.986
32296 measured reflections
2684 independent reflections
2103 reflections with *I* > 2σ(*I*)
*R*_int_ = 0.053

#### Refinement

*R*[*F* ^2^ > 2σ(*F* ^2^)] = 0.067
*wR*(*F* ^2^) = 0.204
*S* = 1.15
2684 reflections
189 parameters
82 restraints
H-atom parameters constrained
Δρ_max_ = 0.35 e Å^−3^
Δρ_min_ = −0.45 e Å^−3^

### 

Data collection: *APEX2* (Bruker, 2005 ▶); cell refinement: *SAINT* (Bruker, 2005 ▶); data reduction: *SAINT*; program(s) used to solve structure: *SHELXS97* (Sheldrick, 2008 ▶); program(s) used to refine structure: *SHELXL97* (Sheldrick, 2008 ▶); molecular graphics: *X-SEED* (Barbour, 2001 ▶); software used to prepare material for publication: *publCIF* (Westrip, 2010 ▶).

## Supplementary Material

Crystal structure: contains datablock(s) global, I. DOI: 10.1107/S1600536811024706/xu5248sup1.cif

Structure factors: contains datablock(s) I. DOI: 10.1107/S1600536811024706/xu5248Isup2.hkl

Supplementary material file. DOI: 10.1107/S1600536811024706/xu5248Isup3.cml

Additional supplementary materials:  crystallographic information; 3D view; checkCIF report

## Figures and Tables

**Fig. 1.**
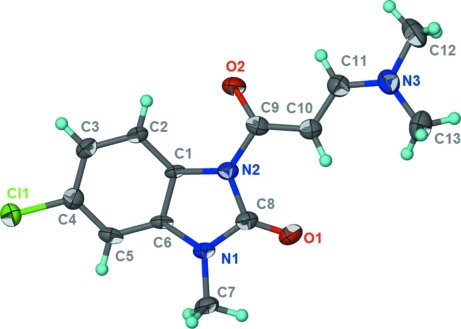
Thermal ellipsoid plot (Barbour, 2001) of C13H14ClN3O2 co-crystal at the 50% probability level; hydrogen atoms are drawn as arbitrary radius. The minor component is not shown.

**Fig. 2.**
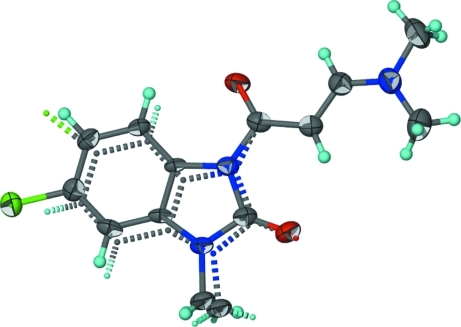
Nearly whole-molecule disorder.

## supplementary crystallographic information

### Comment

In the reaction of 7-chloro-1,5-benzodiazepine-2,4-dione (Mondieig *et al.*, 2007) with *N*,*N*-dimethylformamide/dimethylacetal, the seven-membered ring that is fused with the chlorobenzene ring undergoes a contraction to form five-membered ring, the reaction yielding C_13_H_14_ClN_3_O_2_. The compound is a 4:1 co-crystal whose major component has the chlorine substituent in the 5-position of the benzimidazolone; the minor component has the chlorine in the 6-position (Scheme I, Fig. 1). The crystal structure is better described in terms of nearly 'whole-molecule disorder" (Fig. 2). Interestingly, if the reactant had been unsubstituted 1,5-benzodiazepine-2,4-dione only, the product would have been a single phase only.

### Experimental

7-Chloro-1,5-benzodiazepine-2,4-dione (0.2 g. 0.95 mmol) in *N*,*N*-dimethylformamide-dimethylacetal (2.25 ml) was heated at 373 K for 4 h. The solid was collected and washed with cold dichloromethane. The brown product was recrystallized from petroleum ether.

### Refinement

Carbon-bound H-atoms were placed in calculated positions (C—H 0.93–0.97 Å) and were included in the refinement in the riding model approximation, with *U*(H) set to 1.2–1.5*U*(C).

The chloro-3-methylbenzimidazol-2-one portion is disordered over two positions so that this portion is composed of a 5-chloro-3-methylbenzimidazol-2-one component and a 6-chloro-3-methylbenzimidazol-2-one. The occupancy refined to an 0.816 (5): 0.184 ratio.

The benzene ring was refined as a rigid hexagon of 1.39 Å sides. Pairs of bond lengths (C–Cl, C–N and C–O) were restrained to within 0.01 Å of each other. The temperature factors of the primed atoms were set to those of the unprimed ones, and the anisotropic temperature factors were restrained to be nearly isotropic.

### Crystal data

**Table:**

C_13_H_14_ClN_3_O_2_	*F*(000) = 1168
*M**_r_* = 279.72	*D*_x_ = 1.413 Mg m^−^^3^
Orthorhombic, *P**b**c**a*	Mo *K*α radiation, λ = 0.71073 Å
Hall symbol: -P 2ac 2ab	Cell parameters from 5721 reflections
*a* = 7.3145 (2) Å	θ = 2.9–26.2°
*b* = 14.2903 (3) Å	µ = 0.29 mm^−^^1^
*c* = 25.1512 (6) Å	*T* = 293 K
*V* = 2628.96 (11) Å^3^	Plate, brown
*Z* = 8	0.30 × 0.25 × 0.05 mm

### Data collection

**Table:**

Bruker APEXII diffractometer	2684 independent reflections
Radiation source: fine-focus sealed tube	2103 reflections with *I* > 2σ(*I*)
graphite	*R*_int_ = 0.053
φ and ω scans	θ_max_ = 26.4°, θ_min_ = 4.0°
Absorption correction: multi-scan (*SADABS*; Sheldrick, 1996)	*h* = −8→9
*T*_min_ = 0.918, *T*_max_ = 0.986	*k* = −14→17
32296 measured reflections	*l* = −31→31

### Refinement

**Table:**

Refinement on *F*^2^	Primary atom site location: structure-invariant direct methods
Least-squares matrix: full	Secondary atom site location: difference Fourier map
*R*[*F*^2^ > 2σ(*F*^2^)] = 0.067	Hydrogen site location: inferred from neighbouring sites
*wR*(*F*^2^) = 0.204	H-atom parameters constrained
*S* = 1.15	*w* = 1/[σ^2^(*F*_o_^2^) + (0.092*P*)^2^ + 3.0624*P*] where *P* = (*F*_o_^2^ + 2*F*_c_^2^)/3
2684 reflections	(Δ/σ)_max_ = 0.001
189 parameters	Δρ_max_ = 0.35 e Å^−^^3^
82 restraints	Δρ_min_ = −0.45 e Å^−^^3^

### Fractional atomic coordinates and isotropic or equivalent isotropic displacement parameters (Å^2^)

**Table:**

	*x*	*y*	*z*	*U*_iso_*/*U*_eq_	Occ. (<1)
Cl1	0.52676 (17)	0.77941 (8)	0.11089 (4)	0.0493 (4)	0.816 (5)
O1	0.5389 (12)	0.7581 (9)	0.4050 (3)	0.0492 (11)	0.816 (5)
N1	0.5117 (6)	0.8034 (3)	0.31659 (15)	0.0341 (8)	0.816 (5)
N2	0.6106 (9)	0.6599 (2)	0.33224 (15)	0.0335 (8)	0.816 (5)
C1	0.6049 (4)	0.67203 (15)	0.27723 (8)	0.0252 (8)	0.816 (5)
C2	0.6483 (5)	0.61571 (14)	0.23398 (10)	0.0316 (8)	0.816 (5)
H2	0.6929	0.5555	0.2394	0.038*	0.816 (5)
C3	0.6252 (5)	0.64935 (17)	0.18256 (9)	0.0372 (9)	0.816 (5)
H3	0.6543	0.6117	0.1536	0.045*	0.816 (5)
C4	0.5586 (6)	0.73931 (19)	0.17439 (9)	0.0376 (10)	0.816 (5)
C5	0.5152 (5)	0.79563 (15)	0.21764 (11)	0.0349 (10)	0.816 (5)
H5	0.4706	0.8558	0.2122	0.042*	0.816 (5)
C6	0.5383 (4)	0.76199 (15)	0.26906 (10)	0.0286 (8)	0.816 (5)
C7	0.4443 (8)	0.8980 (3)	0.32573 (18)	0.0418 (12)	0.816 (5)
H7A	0.3302	0.9063	0.3074	0.063*	0.816 (5)
H7B	0.5318	0.9424	0.3126	0.063*	0.816 (5)
H7C	0.4264	0.9076	0.3631	0.063*	0.816 (5)
C8	0.5526 (9)	0.7426 (3)	0.3577 (2)	0.0367 (10)	0.816 (5)
Cl1'	0.6682 (7)	0.6174 (3)	0.13514 (17)	0.0493 (4)	0.184
O1'	0.532 (5)	0.766 (5)	0.4135 (13)	0.0492 (11)	0.184
N1'	0.500 (3)	0.8135 (16)	0.3296 (7)	0.0341 (8)	0.184
N2'	0.609 (4)	0.6657 (9)	0.3400 (7)	0.0335 (8)	0.184
C1'	0.588 (2)	0.6955 (10)	0.2875 (4)	0.0252 (8)	0.184
C2'	0.632 (3)	0.6387 (9)	0.2445 (6)	0.0316 (8)	0.184
H2'	0.6760	0.5785	0.2502	0.038*	0.184 (5)
C3'	0.609 (3)	0.6718 (12)	0.1930 (5)	0.0372 (9)	0.184
C4'	0.542 (3)	0.7617 (13)	0.1845 (5)	0.0376 (10)	0.184
H4'	0.5271	0.7839	0.1500	0.045*	0.184 (5)
C5'	0.499 (3)	0.8186 (9)	0.2275 (7)	0.0349 (10)	0.184
H5'	0.4549	0.8787	0.2218	0.042*	0.184 (5)
C6'	0.5221 (18)	0.7855 (9)	0.2790 (5)	0.0286 (8)	0.184
C7'	0.431 (4)	0.9061 (17)	0.3435 (11)	0.0418 (12)	0.184
H7'A	0.4976	0.9528	0.3241	0.063*	0.184 (5)
H7'B	0.4466	0.9163	0.3810	0.063*	0.184 (5)
H7'C	0.3036	0.9100	0.3347	0.063*	0.184 (5)
C8'	0.548 (3)	0.7466 (13)	0.3669 (11)	0.0367 (10)	0.184
O2	0.6605 (4)	0.50518 (15)	0.32831 (9)	0.0496 (7)	
N3	0.7521 (4)	0.4747 (2)	0.48942 (11)	0.0491 (8)	
C9	0.6537 (4)	0.5735 (2)	0.35780 (13)	0.0369 (8)	
C10	0.6877 (4)	0.5726 (2)	0.41357 (13)	0.0385 (8)	
H10	0.6848	0.6278	0.4331	0.046*	
C11	0.7244 (5)	0.4893 (2)	0.43769 (13)	0.0419 (8)	
H11	0.7309	0.4371	0.4157	0.050*	
C12	0.7832 (7)	0.3809 (3)	0.51016 (17)	0.0679 (13)	
H12A	0.7952	0.3377	0.4811	0.102*	
H12B	0.6816	0.3627	0.5320	0.102*	
H12C	0.8931	0.3803	0.5310	0.102*	
C13	0.7423 (6)	0.5493 (3)	0.52792 (14)	0.0616 (11)	
H13A	0.6539	0.5948	0.5165	0.092*	
H13B	0.8599	0.5786	0.5311	0.092*	
H13C	0.7064	0.5243	0.5618	0.092*	

### Atomic displacement parameters (Å^2^)

**Table:**

	*U*^11^	*U*^22^	*U*^33^	*U*^12^	*U*^13^	*U*^23^
Cl1	0.0640 (7)	0.0447 (6)	0.0391 (6)	−0.0008 (5)	0.0014 (5)	0.0036 (4)
O1	0.0615 (18)	0.041 (3)	0.045 (3)	0.0082 (17)	0.0003 (18)	−0.012 (2)
N1	0.0370 (16)	0.0230 (17)	0.042 (2)	0.0014 (12)	0.0022 (15)	−0.0035 (15)
N2	0.0343 (14)	0.0272 (13)	0.0391 (18)	−0.0006 (12)	−0.0039 (15)	−0.0031 (12)
C1	0.0230 (15)	0.0169 (17)	0.0357 (18)	−0.0020 (14)	0.0010 (14)	−0.0065 (14)
C2	0.0322 (18)	0.0241 (19)	0.039 (2)	−0.0012 (16)	−0.0007 (16)	−0.0060 (14)
C3	0.038 (2)	0.036 (2)	0.038 (2)	−0.0052 (18)	0.0046 (17)	−0.0077 (16)
C4	0.037 (2)	0.038 (3)	0.038 (2)	−0.0075 (19)	0.0018 (17)	−0.0005 (16)
C5	0.0281 (18)	0.0218 (19)	0.055 (3)	−0.0007 (16)	−0.0005 (17)	0.0045 (18)
C6	0.0223 (16)	0.016 (2)	0.047 (2)	−0.0009 (15)	0.0009 (14)	−0.0027 (14)
C7	0.049 (2)	0.0284 (18)	0.048 (3)	0.0036 (16)	0.003 (2)	−0.008 (2)
C8	0.0359 (18)	0.0306 (16)	0.044 (3)	0.0020 (14)	−0.0027 (18)	−0.0055 (16)
Cl1'	0.0640 (7)	0.0447 (6)	0.0391 (6)	−0.0008 (5)	0.0014 (5)	0.0036 (4)
O1'	0.0615 (18)	0.041 (3)	0.045 (3)	0.0082 (17)	0.0003 (18)	−0.012 (2)
N1'	0.0370 (16)	0.0230 (17)	0.042 (2)	0.0014 (12)	0.0022 (15)	−0.0035 (15)
N2'	0.0343 (14)	0.0272 (13)	0.0391 (18)	−0.0006 (12)	−0.0039 (15)	−0.0031 (12)
C1'	0.0230 (15)	0.0169 (17)	0.0357 (18)	−0.0020 (14)	0.0010 (14)	−0.0065 (14)
C2'	0.0322 (18)	0.0241 (19)	0.039 (2)	−0.0012 (16)	−0.0007 (16)	−0.0060 (14)
C3'	0.038 (2)	0.036 (2)	0.038 (2)	−0.0052 (18)	0.0046 (17)	−0.0077 (16)
C4'	0.037 (2)	0.038 (3)	0.038 (2)	−0.0075 (19)	0.0018 (17)	−0.0005 (16)
C5'	0.0281 (18)	0.0218 (19)	0.055 (3)	−0.0007 (16)	−0.0005 (17)	0.0045 (18)
C6'	0.0223 (16)	0.016 (2)	0.047 (2)	−0.0009 (15)	0.0009 (14)	−0.0027 (14)
C7'	0.049 (2)	0.0284 (18)	0.048 (3)	0.0036 (16)	0.003 (2)	−0.008 (2)
C8'	0.0359 (18)	0.0306 (16)	0.044 (3)	0.0020 (14)	−0.0027 (18)	−0.0055 (16)
O2	0.0705 (18)	0.0299 (12)	0.0483 (14)	0.0076 (12)	−0.0090 (12)	−0.0024 (10)
N3	0.0491 (18)	0.0548 (19)	0.0433 (16)	0.0079 (15)	0.0003 (14)	0.0081 (13)
C9	0.0320 (17)	0.0316 (16)	0.0470 (18)	0.0005 (13)	−0.0020 (13)	0.0020 (14)
C10	0.0364 (17)	0.0352 (16)	0.0439 (17)	0.0012 (14)	−0.0004 (14)	−0.0024 (14)
C11	0.0386 (19)	0.0417 (18)	0.0453 (18)	0.0013 (15)	−0.0004 (14)	0.0015 (14)
C12	0.071 (3)	0.074 (3)	0.059 (2)	0.018 (2)	0.011 (2)	0.029 (2)
C13	0.063 (3)	0.080 (3)	0.0422 (19)	−0.003 (2)	−0.0037 (19)	−0.0049 (19)

### Geometric parameters (Å, °)

**Table:**

Cl1—C4	1.713 (2)	N2'—C9	1.430 (9)
O1—C8	1.213 (5)	C1'—C2'	1.3900
N1—C6	1.348 (3)	C1'—C6'	1.3900
N1—C8	1.383 (5)	C2'—C3'	1.3900
N1—C7	1.458 (5)	C2'—H2'	0.9300
N2—C1	1.395 (3)	C3'—C4'	1.3900
N2—C8	1.409 (4)	C4'—C5'	1.3900
N2—C9	1.427 (4)	C4'—H4'	0.9300
C1—C2	1.3900	C5'—C6'	1.3900
C1—C6	1.3900	C5'—H5'	0.9300
C2—C3	1.3900	C7'—H7'A	0.9600
C2—H2	0.9300	C7'—H7'B	0.9600
C3—C4	1.3900	C7'—H7'C	0.9600
C3—H3	0.9300	O2—C9	1.227 (4)
C4—C5	1.3900	N3—C11	1.333 (4)
C5—C6	1.3900	N3—C13	1.442 (5)
C5—H5	0.9300	N3—C12	1.457 (5)
C7—H7A	0.9600	C9—C10	1.424 (5)
C7—H7B	0.9600	C10—C11	1.363 (5)
C7—H7C	0.9600	C10—H10	0.9300
Cl1'—C3'	1.705 (8)	C11—H11	0.9300
O1'—C8'	1.213 (10)	C12—H12A	0.9600
N1'—C6'	1.343 (10)	C12—H12B	0.9600
N1'—C8'	1.385 (10)	C12—H12C	0.9600
N1'—C7'	1.458 (10)	C13—H13A	0.9600
N2'—C1'	1.395 (9)	C13—H13B	0.9600
N2'—C8'	1.410 (10)	C13—H13C	0.9600
			
C6—N1—C8	110.9 (4)	C5'—C4'—H4'	120.0
C6—N1—C7	126.6 (4)	C3'—C4'—H4'	120.0
C8—N1—C7	122.5 (3)	C4'—C5'—C6'	120.0
C1—N2—C8	109.7 (3)	C4'—C5'—H5'	120.0
C1—N2—C9	124.1 (3)	C6'—C5'—H5'	120.0
C8—N2—C9	125.9 (4)	N1'—C6'—C5'	140.1 (15)
C2—C1—C6	120.0	N1'—C6'—C1'	99.9 (15)
C2—C1—N2	134.2 (2)	C5'—C6'—C1'	120.0
C6—C1—N2	105.8 (2)	N1'—C7'—H7'A	109.5
C3—C2—C1	120.0	N1'—C7'—H7'B	109.5
C3—C2—H2	120.0	H7'A—C7'—H7'B	109.5
C1—C2—H2	120.0	N1'—C7'—H7'C	109.5
C4—C3—C2	120.0	H7'A—C7'—H7'C	109.5
C4—C3—H3	120.0	H7'B—C7'—H7'C	109.5
C2—C3—H3	120.0	O1'—C8'—N1'	118 (4)
C5—C4—C3	120.0	O1'—C8'—N2'	133 (4)
C5—C4—Cl1	120.32 (15)	N1'—C8'—N2'	109 (2)
C3—C4—Cl1	119.67 (15)	C11—N3—C13	122.1 (3)
C4—C5—C6	120.0	C11—N3—C12	121.2 (3)
C4—C5—H5	120.0	C13—N3—C12	116.6 (3)
C6—C5—H5	120.0	O2—C9—C10	125.5 (3)
N1—C6—C5	131.0 (3)	O2—C9—N2	115.1 (3)
N1—C6—C1	109.0 (3)	C10—C9—N2	119.3 (3)
C5—C6—C1	120.0	O2—C9—N2'	123.6 (8)
O1—C8—N1	126.9 (8)	C10—C9—N2'	110.9 (8)
O1—C8—N2	128.5 (8)	C11—C10—C9	118.7 (3)
N1—C8—N2	104.6 (4)	C11—C10—H10	120.6
C6'—N1'—C8'	114 (2)	C9—C10—H10	120.6
C6'—N1'—C7'	122.7 (19)	N3—C11—C10	127.0 (3)
C8'—N1'—C7'	123.5 (16)	N3—C11—H11	116.5
C1'—N2'—C8'	99.7 (15)	C10—C11—H11	116.5
C1'—N2'—C9	127.0 (15)	N3—C12—H12A	109.5
C8'—N2'—C9	132.6 (15)	N3—C12—H12B	109.5
C2'—C1'—C6'	120.0	H12A—C12—H12B	109.5
C2'—C1'—N2'	122.3 (12)	N3—C12—H12C	109.5
C6'—C1'—N2'	117.7 (12)	H12A—C12—H12C	109.5
C1'—C2'—C3'	120.0	H12B—C12—H12C	109.5
C1'—C2'—H2'	120.0	N3—C13—H13A	109.5
C3'—C2'—H2'	120.0	N3—C13—H13B	109.5
C2'—C3'—C4'	120.0	H13A—C13—H13B	109.5
C2'—C3'—Cl1'	127.5 (11)	N3—C13—H13C	109.5
C4'—C3'—Cl1'	112.3 (11)	H13A—C13—H13C	109.5
C5'—C4'—C3'	120.0	H13B—C13—H13C	109.5
			
C8—N2—C1—C2	178.9 (2)	Cl1'—C3'—C4'—C5'	−175.6 (11)
C9—N2—C1—C2	−6.1 (7)	C3'—C4'—C5'—C6'	0.0
C8—N2—C1—C6	−1.5 (3)	C8'—N1'—C6'—C5'	180.0 (3)
C9—N2—C1—C6	173.4 (5)	C7'—N1'—C6'—C5'	0.1 (8)
C6—C1—C2—C3	0.0	C8'—N1'—C6'—C1'	−0.1 (5)
N2—C1—C2—C3	179.5 (3)	C7'—N1'—C6'—C1'	−180.0 (5)
C1—C2—C3—C4	0.0	C4'—C5'—C6'—N1'	180.0 (4)
C2—C3—C4—C5	0.0	C4'—C5'—C6'—C1'	0.0
C2—C3—C4—Cl1	−178.8 (3)	C2'—C1'—C6'—N1'	−180.0 (3)
C3—C4—C5—C6	0.0	N2'—C1'—C6'—N1'	0.0 (4)
Cl1—C4—C5—C6	178.8 (3)	C2'—C1'—C6'—C5'	0.0
C8—N1—C6—C5	180.0 (2)	N2'—C1'—C6'—C5'	−180.0 (3)
C7—N1—C6—C5	1.7 (5)	C6'—N1'—C8'—O1'	−180.0 (6)
C8—N1—C6—C1	−2.1 (3)	C7'—N1'—C8'—O1'	−0.1 (10)
C7—N1—C6—C1	179.6 (3)	C6'—N1'—C8'—N2'	0.1 (7)
C4—C5—C6—N1	177.7 (3)	C7'—N1'—C8'—N2'	180.0 (6)
C4—C5—C6—C1	0.0	C1'—N2'—C8'—O1'	−180.0 (7)
C2—C1—C6—N1	−178.2 (2)	C9—N2'—C8'—O1'	9(3)
N2—C1—C6—N1	2.2 (3)	C1'—N2'—C8'—N1'	0.0 (6)
C2—C1—C6—C5	0.0	C9—N2'—C8'—N1'	−172 (2)
N2—C1—C6—C5	−179.6 (2)	C1—N2—C9—O2	−10.2 (7)
C6—N1—C8—O1	−178.3 (4)	C8—N2—C9—O2	164.0 (4)
C7—N1—C8—O1	0.1 (7)	C1—N2—C9—C10	168.9 (4)
C6—N1—C8—N2	1.1 (4)	C8—N2—C9—C10	−17.0 (6)
C7—N1—C8—N2	179.5 (4)	C1—N2—C9—N2'	178 (9)
C1—N2—C8—O1	179.7 (4)	C8—N2—C9—N2'	−8(9)
C9—N2—C8—O1	4.8 (8)	C1'—N2'—C9—O2	−8(2)
C1—N2—C8—N1	0.3 (4)	C8'—N2'—C9—O2	161.0 (11)
C9—N2—C8—N1	−174.5 (5)	C1'—N2'—C9—C10	171.9 (12)
C8'—N2'—C1'—C2'	−180.0 (3)	C8'—N2'—C9—C10	−19 (2)
C9—N2'—C1'—C2'	−8(2)	C1'—N2'—C9—N2	0(7)
C8'—N2'—C1'—C6'	0.0 (5)	C8'—N2'—C9—N2	170 (10)
C9—N2'—C1'—C6'	172 (2)	O2—C9—C10—C11	−2.6 (5)
C6'—C1'—C2'—C3'	0.0	N2—C9—C10—C11	178.5 (4)
N2'—C1'—C2'—C3'	180.0 (3)	N2'—C9—C10—C11	177.1 (12)
C1'—C2'—C3'—C4'	0.0	C13—N3—C11—C10	1.3 (6)
C1'—C2'—C3'—Cl1'	174.9 (13)	C12—N3—C11—C10	177.6 (4)
C2'—C3'—C4'—C5'	0.0	C9—C10—C11—N3	−177.1 (3)
